# The Curious Case of Baby Formula in the United States in 2022: Cries for Urgent Action Months after Silence in the Midst of Alarm Bells

**DOI:** 10.1007/s41055-022-00115-1

**Published:** 2022-12-06

**Authors:** Jinho Jung, Nicole Olynk Widmar, Brenna Ellison

**Affiliations:** Department of Agricultural Economics, Purdue University, 403 West State Street, West Lafayette, IN 47906 USA

**Keywords:** Baby formula shortage, Baby formula shortage, Recall announcement, Social media data, H75, Q18, Y10, Z13

## Abstract

The shortages of baby formula in the US resulting from the voluntary recall of contaminated products and shutdown of manufacturing facility in February led to increases in the national out-of-stock rate of the baby formula from 18 to 70% over the summer of 2022. This study utilizes social media listening and data analysis to examine how online media reactions to the physical shortage changed over time and how the reaction to the shortage differed from to the initial recall announcements. Improved understanding of reactions to emergent issues in foods through this lens may improve communication efficiency to mitigate potential consequences.

## Introduction

An acute shortage of baby formula was still an ongoing issue in the US in the Summer of 2022 (Abrams and Duggan 2022; Doherty et al. 2022; FDA 2022a; New York Post 2022; Samuel et al. 2022). The shortage of physical product resulted from Abbott Nutrition’s voluntary recall of its several brands of powdered formula products due to bacterial contamination from *Cronobacter sakazakii* or *Salmonella* (FDA 2022) and shutdown of their Michigan facility in February, 2022 (Abrams and Duggan 2022). Abbott Nutrition, the largest infant formula manufacturer, provides 40% of the nation’s infant formula (Jaffe 2022) and as a result of its plant shutdown, the national out-of-stock rate for infant formula increased from 18% in January to 43% in the beginning of May, reaching 70% for the week ending May 21^st^ (Bloomberg 2022; Datasembly 2022). The shortage rate spiked over 80% in several states such as California, Missouri, Minnesota, Nevada, Montana, Louisiana, Arizona (Bloomberg 2022; Datasembly 2022). While media outlets provided coverage in May and June 2022 (Bloomberg 2022; Datasembly 2022; The New York Times 2022), the warnings were ample, yet unheeded, months earlier.

The shortage led parents with infants to face uncertainty in safely feeding babies. The pain has been particularly acute for families with low-income and nutritionally vulnerable infants because Abbott Nutrition is the major supplier of infant formula for the government’s Special Supplemental Nutrition Program for Women, Infants, and Children (WIC) (Doherty et al. 2022; The White House 2022). In addition, babies requiring specialty formulas due to allergies, genetic disorders, digestive difficulties, or a variety of other reasons have limited options for replacements of formulas when they are missing from store shelves. Search costs, both in time and money, for parents to search multiple stores challenged vulnerable households for whom transportation, child care, and funds to procure formulas may not be available.

Over the past few months, the federal government and related agencies have diligently worked to resolve the formula shortage. For instance, the federal government cooperated with other infant formula manufacturers to increase production, implemented a process that facilitates the importation of infant formula at the US ports for quick distribution across the US continent, and called on retailers to set limits on the number of purchases to prevent stockpiling (FDA 2022b; The White House 2022).

In addition to the government efforts, recent studies make recommendations to deal with supply chain challenges and shortages in the future, leaning on lessons learned not only in response to recalls but also to other events that may disrupt the supply chain of infant formula, such as the COVID-19 pandemic. Abrams and Duggan (2022) propose a series of interventions to ensure that a similar problem does not occur in the future. They divide the interventions into three themes: 1) public understanding of shortages, 2) prevention of similar events in the future, and 3) support for breastfeeding families (Abrams and Duggan 2022). Their first theme recommends consumers be wary of unauthorized information about alternative formulas in the online space, emphasizing the importance of releasing complete guidance about the recall and safe preparation of homemade formula. The second theme relates to the shortages in the supply system and easier access to alternatives, specifying a need to diversify manufacturers for production of critical/specialty formula and to develop a detailed database of similar products so that families or caregivers can easily identify suitable substitute products. Essentially, the development of a database suggests an intent to reduce search costs for families. Finally, they point out the necessity of more breastfeeding supportive working environments and national policies allowing donor milk (Abrams and Duggan 2022). Supporting breastfeeding is also addressed by others as a long-term means to reduce the demand for commercial baby formula (Doherty et al. 2022; Gill 2022), although there remain unaddressed challenges to breastfeeding for many women and infants alike (e.g., see Dennis 2006; Kelleher 2006; Shah 2013).

To the best of our knowledge, no existing study has examined the formula recall and shortage that followed using online and social media listening and the lens of risk/disaster communication and public policy. The main objective of this study is to identify how online media reacts to recall announcements versus physical shortages and studies the shortage of infant formula through this lens. Social listening and online media analytics can provide insights into real time experiences of large numbers of people in publicizing and then reacting to the recall and shortage.

## Food Recall Announcements, Public Reaction/Awareness to the Recalls, and Risk Management

**Fig. 1:**
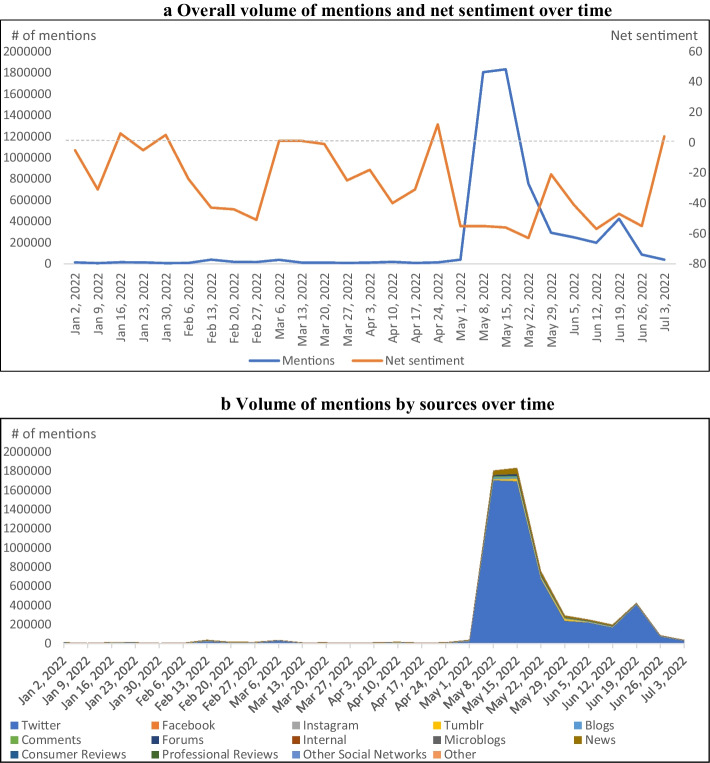
Volume of mentions and net sentiment over time. **a** Overall volume of mentions and net sentiment over time. **b** Volume of mentions by sources over time

The formula supply shortages originated from the voluntary recall in February, 2022. In February, FDA announced the recall and informed consumers about which products should be avoided and how to identify recalled products (FDA 2022c, 2022d). One of the FDA’s updates on February 25^th^ mentioned availability of certain types of infant formula and potential supply chain issues (FDA 2022e). However, search results show that online media, including social media and news, did not react to the FDA’s recall announcements with increased volume in February, but instead rose in total media volume surrounding news of the actual physical shortage issues in May (Fig. 1a), around the moment that the out-of-stock rate of baby formula soared (Bloomberg 2022; Datasembly 2022). The timing and volume of media attention suggests that online and social media did not react until threats – in this case, supply shortfalls – were realized, despite recall announcements sounding the alarm several months earlier. Previous work found that mentions on foodborne illnesses in online space move closely with the CDC’s initial reports of foodborne illness outbreaks, which precede official recalls via FSIS or FDA (Jung et al. 2021). In both cases online media reacts most strongly to the physical threat (illness in the foodborne illness case or shortage in the formula case) rather than the recall announcements, regardless of which comes first.

**Fig. 2:**
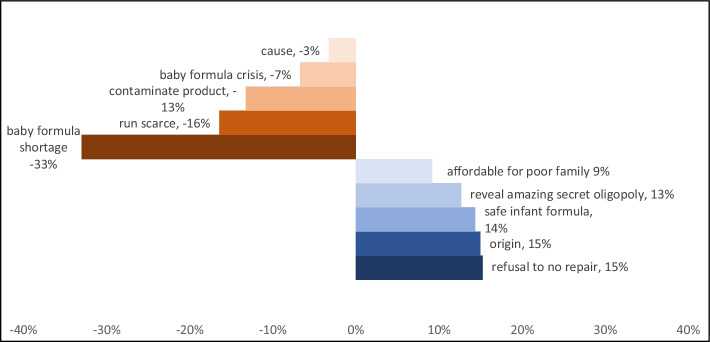
Sentiment drivers for the infant formula shortage search

Online and social media net sentiment can be loosely interpreted as the overall positivity or negativity about a topic or set of search results. Net sentiment is necessarily bounded between -100% and + 100%. The net sentiment about baby formula during the first half of 2022 was mostly negative (Fig. 1a) with the top five negative sentiment drivers identified as *baby formula shortage*, *run scarce*, *contaminate products*, *baby formula crisis*, and *cause* (Fig. 2). Net sentiment plummeted both in February and May despite few mentions posted in February (Fig. 1a), suggesting that at least some people expressed negative sentiment about infant/baby formula early on. Figure 1b illustrates that most of the mentions are posted on Twitter (90% of the total mentions), followed by News (4%) and others. This is consistent with findings from existing studies on crisis management in that social media reacts immediately to crises, such as natural disasters (Widmar et al. 2021, 2022; Houston et al. 2015; Alexander 2014; Nagar et al. 2012; Velev and Zlateva 2012) and foodborne illnesses outbreaks (Jung et al. 2021, 2022).

Another notable online media behavior is that a sudden increase in the mentions in May quickly dissipated within three weeks, despite a continuous rise in the out-of-stock rate over late May through mid-June (Bloomberg 2022; Datasembly 2022; The New York Times 2022). Out-of-stock rates continued to surge even with an increase in importation of infant formula products in June. While beyond the timeframe of study in this analysis and decaying online activities of the public, the U.S. continues to face challenges in infant formula markets as evidenced by the recent additional recall in October of 2022 by Abbott (CNN 2022; CBS news 2022).

This lessening of online media attention over time while the threat is still persistent is consistent with other existing studies for natural disasters such as hurricanes and wildfires (Widmar et al. 2021; Martin et al. 2017) and the ZIKA virus (Widmar et al. 2020a, b). Even in the face of significant threats, public attention dissipates quickly in response to even national scale challenges. Given the importance of public awareness and information about alternative options during the formula shortage (Abrams and Duggan 2022), the quick decay of mentions in online media may leave desperate parents underinformed or misinformed. This could result in the use of unregulated infant formula or over-dilution of formula, which may leave infants exposed to threats, exacerbating the current public health problems.

## Policy Implications

The infant formula situation could have been somewhat mitigated if the supply chain warning in the FDA’s recall announcement would have been heeded by the public, related industries, and the government when the recall was first issued. The Biden administration was not briefed on the formula crisis for weeks after the recall in February and did not know how serious the infant formula shortage would be until April, according to Politico (2022). Further, people in social and online media did not talk about the recall and shortages until May, when they became exposed to the physical product shortages and the higher out-of-stock rates. The lack of timely response, or even timely coverage and educational campaigns to help parents prepare, arguably worsened the panic when physical shortages occurred, seemingly without warning for many people.

Jung et al. (2021) previously studied online and social media responses to food recalls, finding that people immediately react to the initial announcements of foodborne illness outbreaks by CDC more quickly and with more volume of attention than the subsequent FDA and/or FSIS recall announcements. This immediacy of response to CDC illness announcements was posited to help people avoid consuming contaminated food in a timelier manner than recall announcements (Jung et al. 2021).

Efficient communication of recalls and the potential consequences (e.g., shortages) of recalls is critical in the case of infant formula. General recall announcements are designed to cease distribution of contaminated products and prevent consumption (Jung et al. 2021), yet consumers are left with a variety of food options to replace recalled products. Infant formula recalls, on the other hand, are not only about keeping babies from eating contaminated formula, but also about finding safe formula alternatives that are accessible to families. Alternatives can be difficult to find, especially for infants with highly specialized formula needs. Furthermore, accessibility can be particularly challenging given the oligopolistic structure of the formula market, such that when one manufacturer goes down, there are a very limited number of substitute options available to consumers.

Given that FDA was already aware of the potential supply chain issues in February and included availability of certain type of infant formula (FDA 2022e), developing a method of more impactful communication may be helpful for consumers to prepare for future threats. Nudging can be suggested as one method of risk management (Chen et al. 2022; Ohtake 2022; Llopis and Perge 2020; Schmidt and Engelen 2020). In this case, in order to enhance accessibility to recall announcements, sending alerts to individual media such as smartphone may be considered. Considering people’s predictable tendencies to stick to the status quo (Rithalia et al. 2009), it can be suggested that requesting consent for opt-in system of receiving text messages alerting food recall and related potential threats upon opening a new wireless account. To avoid people getting too many alert messages, opt-in process can be more specified upon different situations or user preference. For the baby formula case, parents may be asked to consent to receive alerts upon births of babies at the hospital. This kind of nudging methods is information provision, which allows nudges to be free from such criticisms on nudge as depriving autonomy (Schmidt and Engelen 2020).

## Materials and Method

Among various social listening analytics tools, the Netbase platform was employed for data collection with search key words identified by researchers such as *Baby Formula* and *Infant formula*. The platform is a leader in social media search engines and social media analytics, and many existing studies have employed it (Jung et al. 2021, 2022; Widmar et al. 2020a, 2021, 2022; Mahoney et al. 2020). Netbase provides volumes of mentions, posts, sentiment through natural language processing (NLP), sentiment drivers, top terms, and other information over a certain timeline specified by researchers (Netbase 2015, 2021). Considering Abbott’s voluntary recalls and its announcements on FDA websites in February 2022, the Netbase platform was used to search the past 6 months, from January 1^st^ to July 4^th^, 2022, with the search limited geographically within the US and to posts in English.

Online media sentiment refers to the attitude, opinion, or expression of the general posters towards a topic, event, or phenomenon (Widmar et al. 2020b; Kuttschreuter et al. 2014; Schweidel and Moe 2014). Exploiting NLP, words or terms are categorized into positive (such as “safe infant formula”, “baby formula relief”), neutral, and negative sentiment (such as “baby formula shortage”, “contaminate product”, “baby formula crisis”). The net sentiment is a measurement of comparing positive and negative posts, arriving at a single metric for analysis. The single numeric value of the net sentiment is the total percentage resulting from calculating percentage of positive posts minus the percentage of negative posts, bounded between + 100% and -100% (Netbase 2022; Jung et al. 2021, 2022; Widmar et al. 2021, 2022).
